# Red Ginseng Marc Oil Inhibits iNOS and COX-2 via NFκB and p38 Pathways in LPS-Stimulated RAW 264.7 Macrophages

**DOI:** 10.3390/molecules171213769

**Published:** 2012-11-22

**Authors:** Min-Ji Bak, Soon-Gi Hong, Jong-Won Lee, Woo-Sik Jeong

**Affiliations:** 1 Department of Food & Life Sciences, College of Biomedical Science & Engineering, Inje University, Gimhae 621-749, Korea; Email: redapplemj@hanmail.net; 2 Ginseng Product Research Institute, R&D Headquarters, Korea Ginseng Corp., Daejeon 305-805, Korea; Email: skhong@kgc.or.kr (S.-G.H.); insamlee@kgc.or.kr (J.-W.L.)

**Keywords:** red ginseng marc oil, NFκB, iNOS, COX-2, p38, MAPK, MKK3/6, TAK1, anti-inflammation, chemoprevention

## Abstract

In this study, we investigated the anti-inflammatory effects of red ginseng marc oil (RMO) in the RAW 264.7 macrophage cell line. RMO was prepared by a supercritical CO_2_ extraction of waste product generated after hot water extraction of red ginseng. RMO significantly inhibited the production of oxidative stress molecules such as nitric oxide and reactive oxygen species in lipopolysaccharide (LPS)-activated RAW 264.7 cells. Levels of inflammatory targets including prostaglandin E2, tumor necrosis factor-α, interleukin (IL)-1β and IL-6 were also reduced after the treatment with RMO. In addition, RMO diminished the expressions of inducible nitric oxide synthase and cyclooxygenase 2 at both mRNA and protein levels. Blockade of nuclear translocation of the p65 subunit of nuclear factor κB (NFκB) was also observed after the treatment of RMO. Furthermore, RMO decreased the phosphorylations of p38 mitogen-activated protein kinase (MAPK) and its upstream kinases including MAPK kinases 3/6 (MKK3/6) and TAK 1 (TGF-β activated kinase 1). Gas chromatographic analysis on RMO revealed that RMO contained about 10% phytosterols including sitosterol, stigmasterol and campesterol which may contribute to the anti-inflammatory properties of RMO. Taken together, these results suggest that the anti-inflammatory effect of RMO in LPS-induced RAW 264.7 macrophages could be associated with the inhibition of NFκB transcriptional activity, possibly via blocking the p38 MAPK pathway.

## 1. Introduction

Inflammation, a biological response to physical or chemical injurious stimuli, is closely associated with the release of *pro*-inflammatory mediators such as inducible nitric oxide synthase (iNOS), cyclooxygenase-2 (COX-2), and *pro*-inflammatory cytokines such as interleukins (ILs) and tumor necrosis factor α (TNF-α) [1]. The expression of these inflammation-related genes is controlled by the intracellular signaling pathways such as nuclear factor κB (NFκB) and mitogen-activated protein kinases (MAPKs) at both transcriptional and post-transcriptional levels [2,3].

NFκB is a pivotal transcription factor regulating various genes expression involved in immune and acute phase inflammatory responses [4]. The transcription factor NFκB forms a cytoplasmic complex with its inhibitors, IκBs, under normal physiological conditions. Stimulation of inflammatory cells with lipopolysaccharide (LPS), TNF, irradiation or viral infection results in the activation of Toll-like receptor 4 and downstream inhibitor κB kinases (IKKs) which in turn phosphorylates IκB and leads to NFκB translocation into the nucleus where it regulates transcription of its target genes [5]. Recently, many studies have demonstrated the roles of phytochemicals in anti-inflammatory activity through down-regulation of the NFκB pathway [6,7,8,9].

Activation of the NFκB signaling pathway is closely linked to the activations of MAPKs, which activate downstream transcription factors that promote inflammatory gene expression [10,11]. Furthermore, MAPKs are phosphorylated by upstream MAPK kinases (MAPKKs), which are dual specificity kinases that can phosphorylate threonine and tyrosine residues, and MKKs are in turn activated by MAPK kinase kinases (MAPKKKs), which are related to serine/threonine protein kinases [12]. These activated transcription factors regulate various *pro*-inflammatory cytokines and mediator factors regulate various *pro*-inflammatory cytokines and mediator genes including iNOS, COX-2, interleukin and TNF-α [13,14,15].

Ginseng (*Panax ginseng*) has been used as a traditional remedy in oriental medicine. Ginseng in Korea is classified into three categories, including fresh, white and red ginseng [16]. Red ginseng has been heat steamed and dried. As a consequence of this process, red ginseng undergoes certain biochemical chances and acquires certain pharmacological properties such as antioxidant [9], anti-viral [17], anti-aging [18], anti-depressant [14], anti-obesity [19], anti-carcinogenic [20] and hepatoprotective [21] effects. These beneficial effects of red ginseng have been mostly studied using its water-soluble fractions, probably because red ginseng is widely consumed in the form of hot-water extract or its concentrates. Therefore, we prepared red ginseng marc oil (RMO) by supercritical CO_2_ extraction of waste-products generated after hot water extraction of red ginseng. In recent years, there has been an increasing interest in extracted essential oils and lipid-soluble bioactive compounds from various herbs and plants compared to water-soluble bioactive compounds. Newly discovered properties of essential oils include antibacterial [22], antifungal [23], antiobesity [24], antioxidant [25] and anti-inflammatory activities [26]. The pharmacological properties of essential oils extracted from plants have been the focus of interest from both academia and the pharmaceutical industry. Recently, our research group documented the antioxidant and hepatoprotective effects of RMO in H_2_O_2_-treated HepG2 cell and CCl_4_-treated mice [27]. In the present study, we show that RMO from red ginseng byproducts has anti-inflammatory activity and elucidate its underlying molecular mechanism for the first time in LPS-induced RAW264.7 cells.

## 2. Results and Discussion

### 2.1. Effects of RMO on Cell Viability

**Figure 1:**
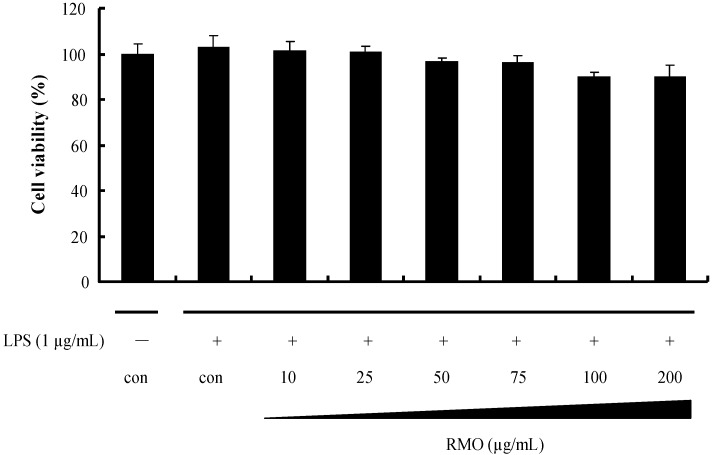
Effects of RMO on LPS-induced RAW264.7 cell viability. Cells were treated with different concentrations of RMO for 48 h, and cell viabilities were determined by MTT assay.

The cytotoxic effects of RMO in RAW 264.7 cells were evaluated using the 3-(4,5-dimethylthiazol-2-yl)-2,5-diphenyltetrazolium bromide (MTT) assay. As shown in Figure 1, RMO did not affect cell viability for 48 h at concentrations up to 200 μg/mL. For the subsequent experiments described in this study, non-toxic dose range (10 to 100 μg/mL) of RMO was used.

### 2.2. RMO Inhibits NO and PGE_2_ Production by Suppressing the Expression of iNOS and COX-2 in LPS-Stimulated RAW 264.7 Cells

**Figure 2:**
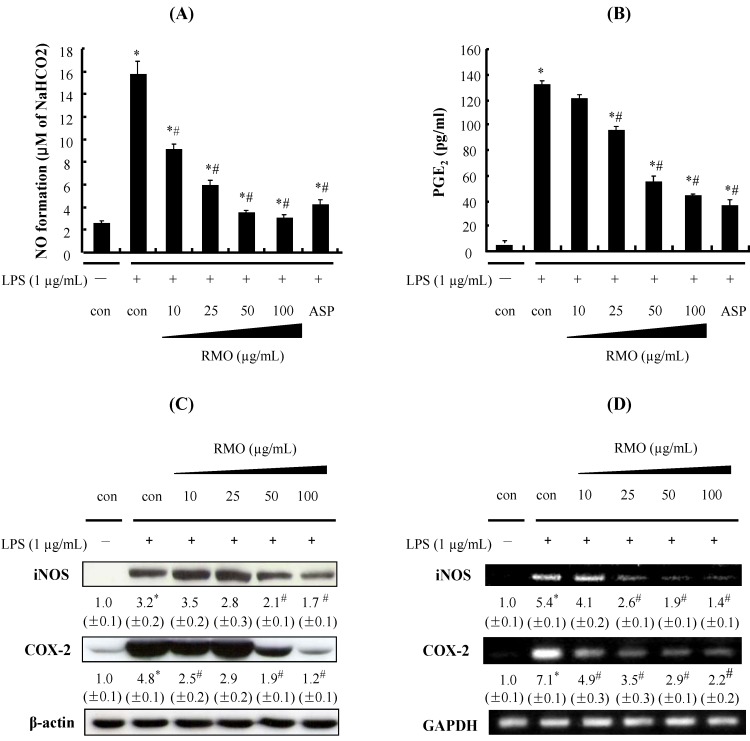
RMO inhibits LPS-induced NO and PGE_2_ productions and iNOS and COX-2 expressions in RAW 264.7 cells. Cells were pretreated with RMO (10, 25, 50 and 100 μg/mL) for 1 h and then exposed to 1 μg/mL of LPS for 24 h. (**A**) The culture medium was subsequently collected, and the nitrite concentration was measured by the Griess reaction. (**B**) Each culture supernatant was subsequently collected, and the amount of PGE_2_ released was measured using the PGE_2_ Parameter Assay Kit. Data are presented as the means ± S.D. of three independent experiments. *, ^#^ Significantly different from the no treatment and LPS-treated control, respectively (*p* < 0.05). Aspirin (ASP) was used as a positive control.(**C**) Treatment of RAW 264.7 cells with RMO for 1 h followed by stimulation with LPS (1 μg/mL) for 24 h in the continued presence of RMO. Then the cells were harvested and whole cell extracts were prepared for Western blot analysis for the indicated proteins. (**D**) The cells were treated with RMO for 1 h, then stimulate with LPS for 24 h, mRNA of iNOS and COX-2 were measured by RT-PCR. The results are expressed as the means ± S.D. for three separate experiments, each with three replicates.

The inflammatory products NO and PGE_2_ can be induced by the expression of iNOS and COX-2, respectively, as a part of the innate immune system, and the inhibition of NO and PGE_2_over-production might be used as a therapeutic tool to treat inflammatory diseases [28]. Since there is a causal relationship between inflammation and cancer, iNOS and COX-2 are often considered as potential molecular targets for chemoprevention [29]. To examine whether RMO can suppress the inflammatory response, the inflammatory responses were induced in RAW 264.7 cells by LPS treatment. As shown in Figure 2A,B, treatment of the cells with LPS dramatically increased NO and PGE_2_ productions; however, RMO significantly inhibited the production of these inflammatory molecules in a dose-dependent manner.

In particular, NO production decreased to near basal levels when the cells were treated with the highest concentration of RMO (100 μg/mL). In addition, RMO treatment at the doses over 50 μg/mL displayed stronger inhibition on NO production than the positive control aspirin, a well-known anti-inflammatory drug. The expressions of inflammatory enzymes iNOS and COX-2 were also strongly induced by LPS treatment while these over-expressions were suppressed by RMO in a concentration-dependent manner both at protein and mRNA levels (Figure 2C,D). Although there seems to be a dose-dependent tendency by RMO treatments in the expressions of these genes, the expression levels of protein and mRNA are not exactly same. The reason for this is not clear at this moment, but there might be an involvement of translational or post-translational modification of these genes and further studies are needed to elucidate the exact mechanisms. Anti-inflammatory agents decreasing NO and PGs productions by simultaneously inhibiting the iNOS and COX-2 genes have been considered to have a potentially therapeutic effect in the treatment of inflammatory and infectious diseases [30,31]. Our results suggest that RMO might inhibit the production of NO and PGE_2_ by down-regulating the expression of iNOS and COX-2, which could be activated by inflammatory stimuli such as LPS.

### 2.3. RMO Inhibits the Protein Expression of Pro-Inflammatory Cytokines in LPS-Stimulated RAW 264.7 Cells

**Figure 3:**
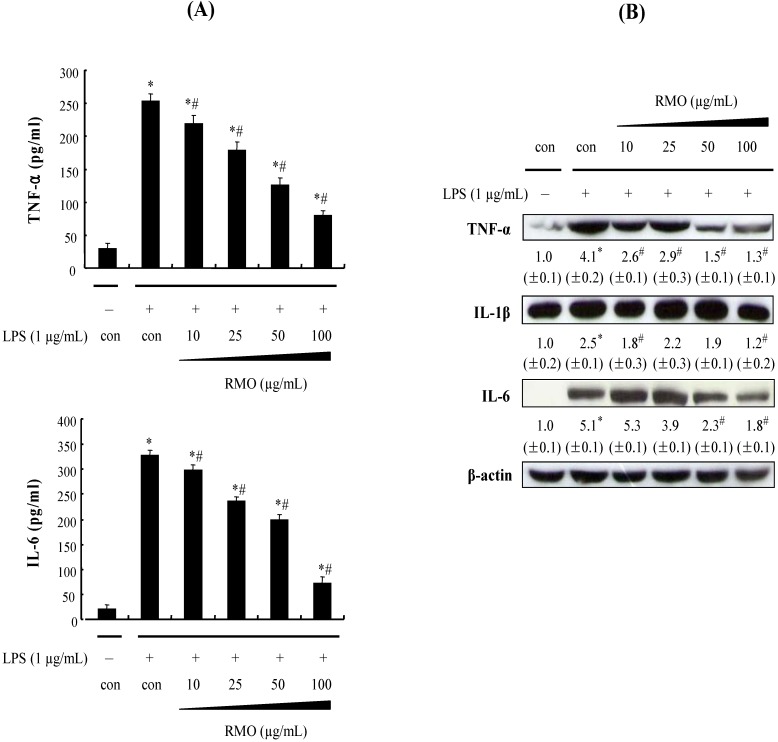
RMO inhibited the production of LPS-induced *pro*-inflammatory cytokine in RAW 264.7 cells. (**A**) RAW 264.6 cells were pretreated with RMO for 1 h, and then stimulated with LPS (1 μg/mL). Culture media were collected after 24 h in order to measured TNF-α and IL-6 concentrations using ELISA. Data are presented as the means ± S.D. of three independent experiments. *, ^#^Significantly different from the no treatment and LPS-treated control, respectively (*p* < 0.05). (**B**) The cells were incubated with RMO in the presence or absence of LPS for 24 h. Then the cells were harvested and whole cell extracts were prepared for Western blot analysis for the indicated proteins. The results are expressed as the means ± S.D. for three separate experiments, each with three replicates.

Certain cytokines such as TNF-α, IL-1β and IL-6 have *pro*-inflammatory effects both *in vitro* and *in vivo* [32,33]. Moreover, TNF-α production is crucially required for the synergistic induction of NO synthesis in IFN-γ- and/or LPS-stimulated macrophages [34]. TNF-α elicits a number of physiological effects such a septic shock, inflammation, cachexia and cytotoxicity; IL-6 is believed to be an endogenous mediator of LPS-induced fever [35]. Therefore, the inhibition of the *pro*-inflammatory cytokines has been identified as targets for anti-inflammatory therapies. Since RMO was found to potently inhibit the *pro*-inflammatory mediators NO and PGE_2_, we investigated its effects on LPS-induced TNF-α, IL-1β and IL-6 releases; this was done by enzyme immunoassay (EIA) and Western blot. As shown in Figure 3A,B, RMO concentration-dependently reduced the production of TNF-α, IL-1β and IL-6 and the corresponding protein expression levels.

### 2.4. RMO Suppresses IKK/IκB/NFκB Signals and NFkB Nuclear Translocation in LPS-Induced in LPS-Stimulated RAW 264.7 Cells

**Figure 4:**
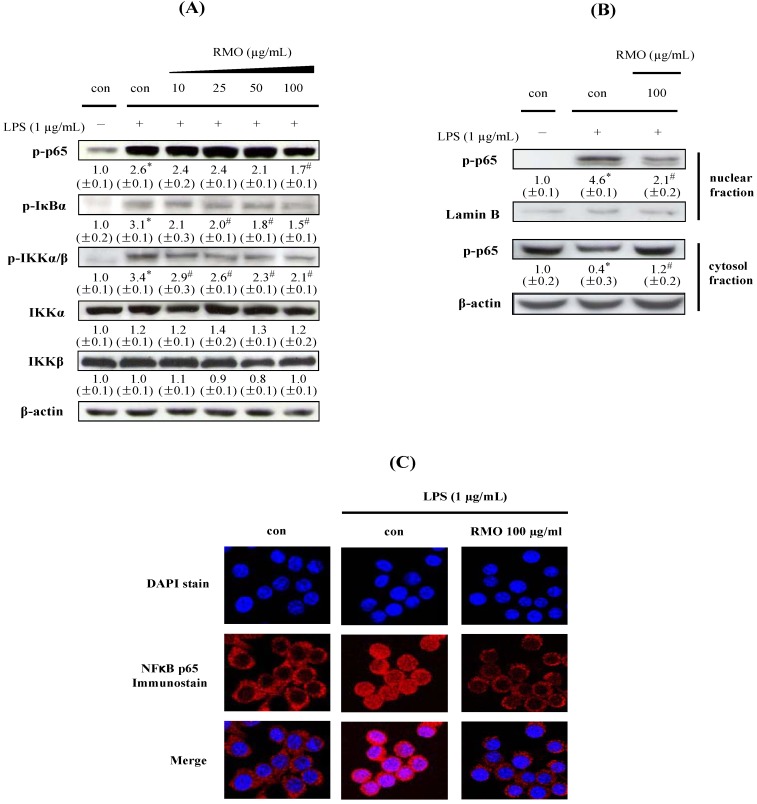
RMO suppresses NFκB activation by blocking NFκB p65 nuclear translocation in LPS-induced RAW 264.7 cells. (**A**) Cell were treated with RMO and stimulated with LPS. Then the cells were harvested and whole cells extracts were prepared for Western blot analysis for the indicated proteins. (**B**) Cells were pretreated with RMO for 1 h and then stimulated with LPS. The cells were harvested at 2 h, and then prepared nuclear extracts for the detection of phosphorylated NFκB p65 subunit and cytosolic extracts for the detection of phosphorylated IκBα by Western blot. (**C**) Cell were pretreated with RMO (100 μg/mL) for 1 h followed by stimulation with LPS for 2 h. Samples were stained by anti-p65 antibody and DAPI then prepared for confocal microscopy analysis. The results are expressed as the means ± S.D. for three separate experiments, each with three replicates. *, ^#^ Significantly different from the no treatment and LPS-treated control, respectively (*p* < 0.05).

Extracellular stimuli including LPS induce IKK/IκB/NFκB pathway to stimulate the production of inflammatory cytokines and *pro*-inflammatory enzymes [36,37,38]. iNOS and COX-2 genes are known to contain NFκB-binding sites in their promoter region [39]. Under unstimulated condition, NFκB is sequestered in the cytosol, associated with the inhibitory IκB protein. The activation of IKKs in response to extracellular stimuli leads to rapid phosphorylation and degradation of IκB, which makes NFκB translocate to the nucleus where it regulates gene transcription [40,41,42]. We examined whether the inhibitory effects of RMO on the production of inflammatory markers including NO, PGE_2_, TNF-α, IL-1β and IL-6 are related to its blockade of the NFκB signaling pathway. Thus, we first assessed regulatory ability of RMO on the phosphorylation of p65 and IκBα. As illustrated in Figure 4A, LPS strongly induced the phosphorylation of p65 and IκBα whereas RMO dose-dependently inhibited these phosphorylations. Phosphorylation of the upstream kinase IKKα/β was also similarly diminished by RMO treatment but the protein level of IKKα/β was not affected by RMO. Therefore, RMO seems to reduce the production of inflammatory markers through inhibiting phosphorylations of IKK/IκB/NFκB signaling pathways.

We next investigated whether RMO could inhibit the nuclear translocation of p65 after its release from IκB using Western blot and confocal microscopy analyses. Upon exposure of cells to LPS, p-p65 was accumulated in the nuclear fraction whereas the nuclear accumulation of p-p65 was reduced to less than half after RMO treatment (Figure 4B). For the cytosolic fractions, the results were opposite. These results were confirmed by immunofluorescence staining assay and the results are displayed in Figure 4C. Cytoplasmic retention of p-p65 was observed in cells treated with RMO, while translocation of p-p65 into the nucleus was observed in LPS-induced cells. Taken together, these results clearly demonstrate that RMO inhibits NFκB activation by preventing the LPS-stimulated nuclear translocation of p-p65 in RAW 264.7 cells. Many studies have suggested that certain phytochemicals could suppress inflammatory responses by regulating the NFκB pathway [43,44,45]. These findings concur with our finding that the transcriptional inhibition of *pro*-inflammatory mediators by RMO is associated with the IKK/NFκB signal pathway.

### 2.5. RMO Inhibits Phosphorylation of MAPK, MAPKK, and MAPKKK in LPS-Stimulated RAW 264.7 Cells

**Figure 5:**
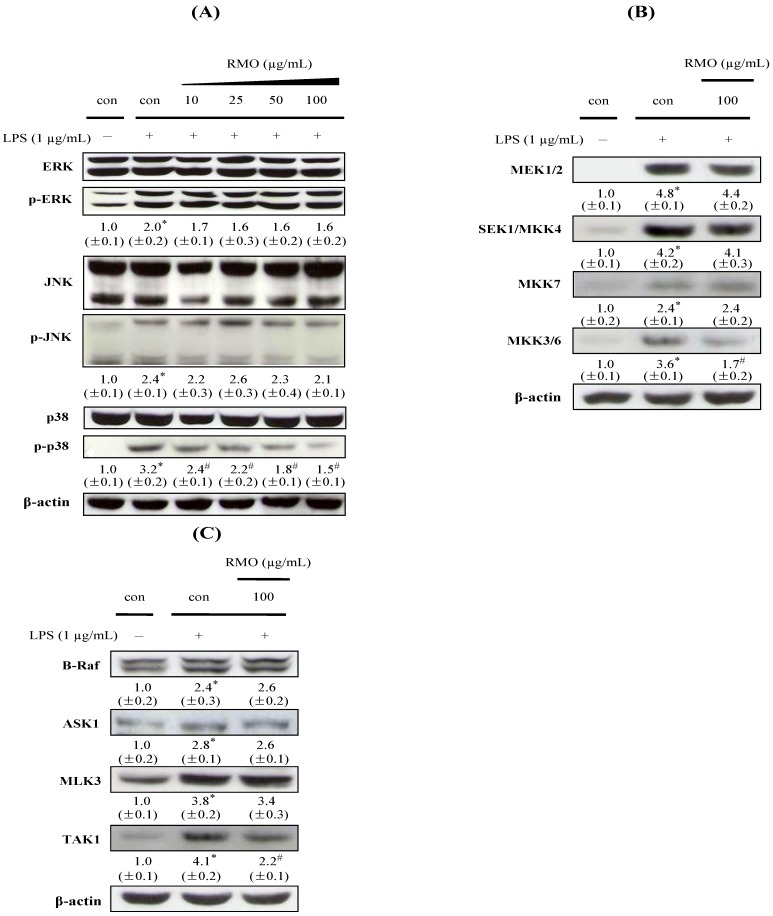
Effects of RMO on MAPK, MAPKK and MAPKKK phosphorylation in LPS-induced RAW 264.7 cells. Cells were treated with RMO for 1 h and stimulated with LPS (**A**) for 1 h, (**B**) 30 min, and (**C**) 15 min. Cell lysates were then subjected to Western blot analysis for the indicated proteins. The results are expressed as the means ± S.D. for three separate experiments, each with three replicates.

Various intracellular pathways are involved in inflammatory actions through up-regulating the production of major mediators on inflammation [46]. Three families of MAPKs including ERK, JNK and p38 play critical roles in cell growth regulation and differentiation and in the control of cellular responses to cytokines and stressors [47]. MAPK phosphorylation activates the transcription of NFkB-mediated *pro*-inflammatory cytokines [48]; Therefore, MAPKs are often considered as molecular targets for the development of novel anti-inflammatory phytochemical. We investigated MAPKs phosphorylations in the presence or absence of RMO in LPS-stimulated cells (Figure 5A). The phosphorylations of ERK, JNK, and p38 were elevated when cells treated with LPS alone. RMO exclusively repressed p38 phosphorylation but did not affect ERK and JNK phosphorylations. p38 pathway inhibition has been reported to decrease *pro*-inflammatory cytokines production in monocytes/macrophages via transcriptional and post-transcriptional regulation [49].

**Figure 6:**
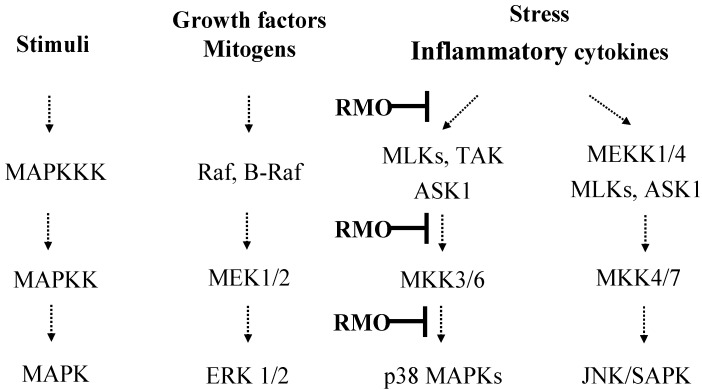
Schematic diagram illustrating the MAPK signaling cascades involved in RMO’s inhibition of LPS-induced inflammation in RAW 264.7 cells.

Furthermore, the activation of MAPKs is known to require both tyrosine and theronine phosphorylation by the activated MAPKKs (MEK1/2, SEK1/MKK4, MKK7 or MKK3/6). Among the MAPKs, p38 MAPK is activated by dual phosphorylation on Thr180 and Tyr182 by upstream MAPK kinases: MAP2K6 or MAP2K3 (MKK3/6), which are activated by upstream MAPKKKs, and stimulated by a variety of stimuli. A MAPKK-independent mechanism of p38 activation involves transforming growth factor-β-activated protein kinase 1 (TAK1)-binding protein 1 (TAB1) [50,51]. To investigate the upstream kinases of p38 MAPK, we examined the modulatory effects of RMO on the phosphorylations of MAPKKs (MKK3/6, MEK1/2, MKK4, and MKK7) and MAPKKKs (TAK1, B-Raf, ASK1, and MLK3). RMO markedly suppressed the phosphorylations of MKK3/6 and TAK1 (Figure 5B,C), suggesting an involvement of RMO in TAK to MKK3/6 to p38 signaling pathways (Figure 6). Overall, our results imply that RMO might exert its anti-inflammatory ability through inhibiting p38 pathway which leads to NFκB inactivation and thereby suppressing expressions of inflammatory target proteins including proinflammatory cytokines and enzymes. Similar results have been reported with chemopreventive phytochemicals such as stercurensin [15], quercetin [52] and carnosol [53] which inhibit *pro*-inflammatory enzymes by targeting MAPK signaling pathways, including the p38 kinase pathway.

### 2.6. Phytosterols in RMO

Phytosterols are plant sterols that are found in seeds, roots, stems, branches, leaves and blossoms of various plants, including medicinal herbs, edible plants, shrubs and trees [54]. The three major phytosterols in plants include sitosterol, campesterol and stigmasterol [55]. It has been widely reported that phytosterols lower serum cholesterol level in animals and humans [54]. Besides the hypocholesterolemic effects, phytosterols have recently been reported to have anti-inflmmatory properties through the inhibition of proinflammatory cytokines including IL-6 and TNF-α [55]. As a part of investigations on active ingredients in RMO, we analyzed the content and composition of RMO using gas chromatography (GC) analysis. As shown in Table 1, RMO contained about 108 mg of phytosterols per g RMO and sitosterol was by far the most abundant phytosterol which accounts for 84% of total phytosterols in RMO. So far, only a few studies have reported phytosterols in ginseng. Sitosterol has been found from the fruit of ginseng [56]. Beveridge *et al*. have reported phytosterol content in the seed oil of American ginseng [57]. In the ginseng seed oil, sitosterol was the most abundant phytosterol followed by stigmasterol and campesterol. There has been no report on the phytosterol content or composition of red ginseng. Although there have been positive correlations between phytosterols and anti-inflammation, it is not clear whether the anti-inflammatory properties of RMO come from its phytosterols at this moment. More studies on the anti-inflammatory mechanisms of RMO phytosterols and studies on the presence of other possible active ingredients should be followed. Volatile compounds profiles, other than the phytosterols, of RMO are under investigation and to be reported separately.

**Table 1:** Phytosterol contents in red ginseng mark oil.

Phytosterol	Content (mg/g RMO)
Campesterol	3.9 ± 0.06
Stigmasterol	13.7 ± 0.27
Sitosterol	90.3 ± 2.58
Total	107.9 ± 2.91

## 3. Experimental

### 3.1. Chemicals

LPS (Escherichia coli O127:B8), Triton X-100 and all other chemicals were purchased from Sigma Chemical Co. (St. Louis, MO, USA).

### 3.2. Preparation of RMO

The supercritical CO_2_ extract preparation was performed according to our previous report with some modifications [27]. In brief, dried red ginseng byproduct powder was placed into the extraction vessel of a pilot-scale supercritical fluid extraction system (Ilshin Autoclave Co., Ltd., Daejeong, Korea). Extractions with supercritical CO_2_ were operated at 6,500 psi (relative to 450 bar) in combination with temperature at 65 °C. Extracted constituents were collected in a vial that was prefilled with a trapping solvent and maintained at 4 °C during the extraction step.

### 3.3. Cell Cultures

RAW264.7 murine macrophage cells were purchased from American Type Culture Collection (ATCC, Rockville, MD, USA). Cells were cultured at 37 °C and 5% CO_2_ in Dulbecco’s modified Eagles’s medium (DMEM) containing 10% FBS, 100 units/mL penicillin, 100 μg/mL streptomycin (Hyclone, Logan, UT, USA).

### 3.4. Analysis of Cell Viability

The MTT [3-(4,5-dimethylthioazol-2-yl)-5-(3-carboxy-methoxyphenyl)-2-(4-sulfophenyl)-2H-tetrazolium, inner salt] assay was performed with a CellTiter 96 aqueous nonradioactive cell proliferation assay kit (Promega Corp., Madison, WI, USA) according to the manufacturer’s instructions. Briefly, the cells were plated on 24-well plates at a density of 1 × 10^5^ cells/well. After 24 h of incubation, the cells were treated with different doses of each sample for 48 h. Then, media were removed, and culture media containing MTS and phenazine methosulfate solution were added. After 1 h, the absorbance was measured at 490 nm with a PowerWave XS microplate reader (BioTek Instruments, Inc., Winooski, VT, USA). The data was expressed as percent cell viability compared to the vehicle control.

### 3.5. Measurement of Nitrite Oxide Formation

Nitrite, as an indicator of NO synthesis, was determined in cell culture supernatants by the Griess reaction. Briefly, RAW 264.7 cells were plated at 1 × 10^5^ cells/well in 24-well plates and incubated at 37 °C for 24 h and then treated with RMO with LPS. After incubation of cells for 24 h, the supernatants (0.1 mL) were added to a solution of 0.1 mL Griess reagent (1% sulfanilamide and 0.1% naphthyl ethylene diaminedihydrochloride in 5% H_3_PO_4_) to form a purple azo dye. Using NaNO_2_ to generate a standard curve, nitrite production was measured by spectrophotometry at 550 nm using a microplate reader (BioTek Instruments, Inc.).

### 3.6. Measurement of PGE_2_ Production

The amount of PGE_2_ produced from endogenous arachidonic acid was measured using a PGE_2_ Parameter Assay Kit (R&D Systems, Minneapolis, MN, USA). RAW264.7 cells (1 × 10^5^ cells/well) were treated with different concentration of RMO for 1 h and stimulated with LPS for 24 h, the conditioned media was collected to perform PGE_2_ enzyme immune-metric assay according to the manufacturer’s protocol. The concentration of PGE_2_ was calculated according to the equation obtained from the standard curve plot using PGE_2_ standard solution in the EIA kit.

### 3.7. Measurement of TNF-α and IL-6 Production

RAW 264.7 cells were plated at 1 × 10^5^ cells/well, and stimulated with LPS for 24 h in the presence or absence of RMO. Culture supernatants were collected and the amount of TNF-α and IL-6 was determined by EIA using a protocol supplied by Amersham (Amersham Pharmacia Biosciences, Piscataway, NJ, USA).

### 3.8. Total RNA Extraction and Reverse Transcriptase (RT)-PCR Analysis

After LPS stimulation (1 μg/mL) of RMO-exposed cells for 24 h, total RNA was isolated using TRIzol reagent (Invitrogen, Carlsbad, CA, USA) and reverse transcribed into cDNA using SuperscriptTM RNase H reverse transcriptase (Invitrogen) according to the manufacturer’s recommendations. Subsequent PCR analysis was carried out with aliquots of the cDNA preparation using a PCR System (Corbett Research, Sydney, Australia). The PCR conditions were as follows: predenaturation at 94 °C for 30 s, annealing at 55 °C (COX-2 and GAPDH) or 59 °C (iNOS) for 30 s and extension at 72 °C for 30 s. The PCR products were visualized in 2% agarose gels and documented using a Gel-Doc EQ system (Bio-Rad, Hercules, CA, USA). The primer sequences were as follows: mouse iNOS, 5'-GCC TTC AAC ACC AAG GTT GTC TGC A-3' (sense), 5'-TCA TTG TAC TCT GAG GGC TGA CAC A-3' (anti-sense); mouse COX-2, 5'-CTG GTG CCT GGT CTG ATG ATG-3' (sense), 5'-GGC AAT GCG GTT CTG ATA CTG-3' (anti-sense); mouse GAPDH (as an internal control for PCR), 5'-CAA TGC CAA GTA TGA TGA CAT-3' (sense), 5'-CCT GTT ATT ATG GGG GTC TG-3' (anti-sense).

### 3.9. Preparation of Whole-Cell, Cytoxolic, and Nuclear Extracts

RAW 264.7 cells grown at 2 × 10^5^ cells/well in 6-well plates were stimulated with RMO for 1 h following pretreatment in the presence of absence of 1 μg/mL LPS for various time periods. Whole-cell extracts were then prepared according to the manufacturer’s instructions using Cell lysis Buffer containing 20 mM Tris (pH 7.5), 135 mM NaCl, 2 mM EDTA, 2 mM DTT, 25 mM β-glycerophosphate, 2 mM sodium pyrophosphate, 10% glycerol, 1% Triton X-100, 1 mM sodium orthovanadate, 10 mM NaF, 10 μg/mL aprotinin, 10 μg/mL leupeptin, and 1 mM PMSF. The cytosolic and nuclear extracts were prepared as above using a kit (Pierce Biotechnology Inc, Rockford, IL, USA). The protein content was quantified by absorbance at 590 nm according to the BCA protein assay (Pierce Biotechnology).

### 3.10. Western Blot Analysis

Proteins (whole-cell extracts: 30 μg/lane, nuclear extracts: 10 μg/lane, cytosolic extracts: 30 μg/lane) were separated by electrophoresed on a 10% SDS-polyacrylamide gel, and transferred to PVDF membranes with a semidry transfer system (Bio-Rad). The membranes were blocked with 5% nonfat milk in PBST with 0.1% Tween 20 for 1 h at room temperature, and then incubated overnight with primary antibodies. After hybridization with primary antibodies, the membrane was washed five times with PBST for 5 min, then incubated with horseradish peroxidase-conjugated secondary antibody for 1 h at RT and washed five times with PBST for 5 min. Final detection was performed with Western Blotting Luminol reagents (SantaCruz Biotechnology, Santa Cruz, CA, USA). Monoclonal antibodies against IKK β, p-IκBα, IκBα, p-NFκB-p65, and ERK and polyclonal antibodies against TNF-α, NFκB-p65, p-IKK α/β, p-EKR, p-p38, p38, p-SAPK/JNK, SAPK/JNK, IKKα, p-MEK1/2, p-MKK3/MKK6, p-B-Raf, MLK3, SEK1/MKK4, p-MKK7, p-ASK1 and p-TAK1 were purchased from Cell Signaling Technology, Inc. (Beverly, MA, USA). The polyclonal antibodies against iNOS, COX-2, IL-1 β, IL-6, Lamin B and β -actin were purchased from Santa Cruz Biotechnology, Inc.

### 3.11. Confocal Microscopy Analysis

RAW264.7 cells were planted on the coverglass bottom dishes. After 24 h, the cells (2 × 10^5^cells/well) were pretreated with 100 μg/mL RMO for 2 h and then stimulated with 1 μg/mL LPS for 1 h. The cells were fixed with 4% paraformaldehyde for 10 min, permeabilized with 0.2% Triton X-100 for 10 min, blocked with 3% BSA in PBS for 2 h, incubated with an anti-p65 primary antibody at room temperature for 2 h, sequentially incubated with an Alexa Fluor 555-conjugated secondary antibody (Cell Signaling) at room temperature for 2 h in the dark, and finally incubated with 1 μg/mL of 4',6'-diamidino-2-phenylindole (DAPI) at room temperature for 20 min in the dark. Images were obtained using a LSM 510 laser confocal microscope (Zeiss, Jena, Germany).

### 3.12. Analysis of Phytosterol Content

Ten g of RMO were saponified in a round-bottom flask containing 0.1 N ethanolic KOH (10 mL) and ethanol (40 mL). The mixture was refluxed for 1 h on a heating mantle at 95 °C. After saponification, the mixture was cooled to room temperature, mixed with saturated NaOH solution (50 mL) and transferred to a separatory funnel. The unsaponifiables were extracted 4 times with 100-mL portions of hexane. The combined hexane extracts were concentrated to dryness using a rotary evaporator and 25 mL of an internal standard solution (5α-cholestane, 1 mg/mL) was added to the concentrate. The unsaponifiables were analyzed by an Agilent 6890 gas chromatograph (Agilent Technologies, Santa Clara, CA, USA) equipped with a DB5 MS column (Agilent) and a flame ionization detector. The column temperature was programmed from 200 °C for 2 min, 200 °C to 300 °C at 5 °C/min, and held for 5 min. Nitrogen gas was the carrier gas at a flow rate of 3 mL/min and the spilt ratio was 10:1. The injection port and the detector temperatures were 270 °C and 300 °C, respectively.

### 3.13. Statistical Analysis

The data were expressed as mean ± S.D. values. The values were compared with the control using analysis of variance followed by unpaired Student’s t tests. A *p* value of <0.05 was considered significant.

## 4. Conclusions

RMO suppressed the LPS-stimulated inflammatory responses including NO and PGE2 generation, secretion of *pro*-inflammatory cytokines of TNF-α, IL-1β and IL-6 and the expression of *pro*-inflammatory mediators such as iNOS and COX-2 in RAW264.7 macrophages. Furthermore, RMO enhanced the inhibitory effects on inflammation via inactivation of NFκB and p38/ MKK3/6/TAK1 signaling pathways. RMO contained about 10% phytosterols including sitosterol, stigmasterol and campesterol which may contribute to the anti-inflammatory properties of RMO. Taken together, our data suggest that RMO might exert its anti-inflammatory ability through inhibiting p38 pathway which leads to NFκB inactivation and thereby suppressing expressions of inflammatory target proteins including proinflammatory cytokines and enzymes. Our findings suggest for the first time the potential of red ginseng marc oil as a potent natural anti-inflammatory agent.
